# Pediatric acute asthma scoring systems: a systematic review and survey of UK practice

**DOI:** 10.1002/emp2.12083

**Published:** 2020-06-02

**Authors:** Jerry Chacko, Charlotte King, David Harkness, Shrouk Messahel, Julie Grice, John Roe, Niall Mullen, Ian P. Sinha, Daniel B. Hawcutt, PERUKI

**Affiliations:** ^1^ School of Medicine University of Liverpool Liverpool UK; ^2^ Department of Women's and Children's Health University of Liverpool Liverpool UK; ^3^ Royal Liverpool and Broadgreen University Hospital Trust Liverpool UK; ^4^ National Institute for Health Research Alder Hey Clinical Research Facility Alder Hey Children's Hospital Liverpool UK; ^5^ Emergency Department Alder Hey Children's Hospital Liverpool UK; ^6^ Darwin Emergency Department Darwin Northern Territory Australia; ^7^ Paediatric Emergency Medicine Sunderland Royal Hospital Sunderland UK; ^8^ Department of Respiratory Medicine Alder Hey Children's Hospital Liverpool UK

**Keywords:** asthma, emergency department, pediatrics, severity score, systematic review

## Abstract

**Background:**

Acute exacerbations of asthma are common in children. Multiple asthma severity scores exist, but current emergency department (ED) use of severity scores is not known.

**Methods:**

A systematic review was undertaken to identify the parameters collected in pediatric asthma severity scores. A survey of Paediatric Emergency Research in the United Kingdom and Ireland (PERUKI) sites was undertaken to ascertain routinely collected asthma data and information about severity scores. Included studies examined severity of asthma exacerbation in children 5–18 years of age with extractable severity parameters.

**Results:**

Sixteen articles were eligible, containing 17 asthma severity scores. The severity scores assessed combinations of 15 different parameters (median, 6; range, 2–8). The most common parameters considered were expiratory wheeze (15/17), inspiratory wheeze (13/17), respiratory rate (10/17), and general accessory muscle use (9/17). Fifty‐nine PERUKI centers responded to the questionnaire. Twenty centers (33.1%) currently assess severity, but few use a published score. The most commonly recorded routine data required for severity scores were oxygen saturations (59/59, 100%), heart rate, and respiratory rate (58/59, 98.3% for both). Among well‐validated scores like the Pulmonary Index Score (PIS), Pediatric Asthma Severity Score (PASS), Childhood Asthma Score (CAS), and the Pediatric Respiratory Assessment Measure (PRAM), only 6/59 (10.2%), 3/59 (5.1%), 1/59 (1.7%), and 0 (0%) of units respectively routinely collect the data required to calculate them.

**Conclusion:**

Standardized published pediatric asthma severity scores are infrequently used. Improved routine data collection focusing on the key parameters common to multiple scores could improve this, facilitating research and audit of pediatric acute asthma.

## 1 INTRODUCTION

Asthma is one of the most common childhood diseases, affecting children of all ages.^1^ Currently, there are approximately 1.1 million children and young people affected by asthma in the United Kingdom.^1^ An exacerbation of asthma can vary in severity from a mild cough and wheeze, to severe breathlessness that can be life‐threatening.^2^ Exacerbations are a common reason for attendance at an emergency department.^3^, ^4^ In the last few years, there has been an increase in UK pediatric asthma admission rates^4^, ^5^; however, the reasons for this increase are not fully understood.

Within the United Kingdom, there is an inequality in the distribution of asthma prevalence and severity in children and young people, with those from more deprived backgrounds suffering from increased frequency and severity of disease.^6^, ^7^ Around the country, there is 25‐fold variation in the pediatric asthma admission rates.^7^ Local authorities in the North West of England have some of the highest rates of emergency admissions for childhood asthma in the United Kingdom.^8^ There may be different factors that have influenced this such as entrenched inequality, access to healthcare, inappropriate admission, or under‐treatment of asthma. However, to fully comprehend the reason, an understanding of the severity of asthma patients who present to EDs needs to be included.

ED staff routinely assess asthma severity when seeing a child, but to facilitate comparisons within and between units, and for research purposes, scoring systems have been designed to provide objective assessments of how unwell individuals are. Each severity score assesses a series of different parameters that alter in an acute asthma attack. There is no single clinical sign that indicates the degree of exacerbation, rather a collection of different symptoms and signs that point to the severity.^9^ Tools to help assess the severity of asthma can provide additional information regarding need for hospitalization or safety of discharge. Historically, failure to assess severity was considered a factor that contributed to mortality.^10^

The current British Thoracic Society/Scottish Intercollegiate Guideline Network (BTS/SIGN) guidelines^2^ categorize the severity of asthma exacerbations into 3 main domains: moderate, severe, and life threatening. Each domain contains different parameters that contribute to degree of severity. Although this scoring system can be used to assess overall severity at presentation, it does not numerically quantify severity, thus limiting its use in audit or research.

Current practice with regard to collection of data relating to, and use of, pediatric asthma severity scores across the United Kingdom and Ireland is not known. The Paediatric Emergency Research in the United Kingdom and Ireland (PERUKI) group^11^ collaboratively undertake pediatric emergency medicine research to improve care of sick and injured children. This group is optimally placed across the United Kingdom and Ireland to assess current practice.

The aim of this study is therefore to identify and extract the parameters used in pediatric asthma severity scores. The data will then be used in a survey to assess current practice with regard to data collection and use of asthma severity in pediatric populations attending ED in the UK and Ireland.

## 2 METHODOLOGY

### 2.1 Study design and setting

To undertake a systematic review of studies that assessed the severity of asthma exacerbations in the pediatric population, defined as children and young people 5 to 18 years of age, was conducted.

### 2.2 Information sources and search strategy

Electronic databases, MEDLINE, CINAHL, and Web of Science were searched up to December 2017 to identify relevant studies. There were no date or language restrictions, the search terms used were based upon 3 main terms; “asthma,” “pediatric,” and “severity score.” See Supporting Information File S1 for full list of search terms.

### 2.3 Inclusion criteria and study selection

The term “severity score,” defined any dyspnea score that was developed to assess the severity of asthma exacerbations, comprises at least 2 different parameters. The “severity score” must have had a numeric value associated with each measured parameter to allow variation in the severity to be assessed.

Articles that described or used a severity score that could be used to measure asthma exacerbations, in patients from 5 to 18 years of age, were included. Articles that assessed dyspnea in relation to other conditions such as bronchiolitis and rhinitis and articles that used a predictive score (looked at the need for hospitalization) were excluded (see Supporting Information File S2 for inclusion and exclusion criteria). Additional papers were located through the analysis of included full‐text screened papers by examining their references to see if additional studies may be eligible for inclusion.

### 2.4 Data extraction

Reviewers (JC and DH) independently screened the titles and abstracts using the pre‐specified criteria; full‐texts of eligible articles were then reviewed to assess eligibility of inclusion. Disagreements on the eligibility of articles were resolved by consensus. Data were extracted into a pre‐defined table.

**THE BOTTOM LINE**

There are many published asthma severity scores for children and young people attending hospital. This study examined the factors used to create these scores, the current use of the scores, and UK emergency department preferences for such scores, to facilitate future data collection and comparative research between the scoring systems.

### 2.5 Study outcomes

The pre‐specified primary outcome were the parameters used to generate each asthma severity score. Secondary outcomes were the number of severity scores that used each specific parameter, and the number of children that each severity score was scored against. Because this study did not involve the synthesis of outcome of new data, the methodological quality of the included studies were not conducted because it was unnecessary.

#### 2.5.1 Survey of UK practice among PERUKI sites

This multi‐center cross‐sectional online survey was delivered using SurveyMonkey (https://www.surveymonkey.com/) between December 19, 2018 and January 30, 2019 across Paediatric Emergency Research in the United Kingdom & Ireland (PERUKI)^12^; 1 nominated clinician per site was asked to respond. The survey was developed iteratively by the study team based feedback from pilot testing. The questionnaire is available in Supplementary File 3. Regular reminders were sent from the PERUKI team to improve response rate.

## 3 RESULTS

**FIGURE 1:**
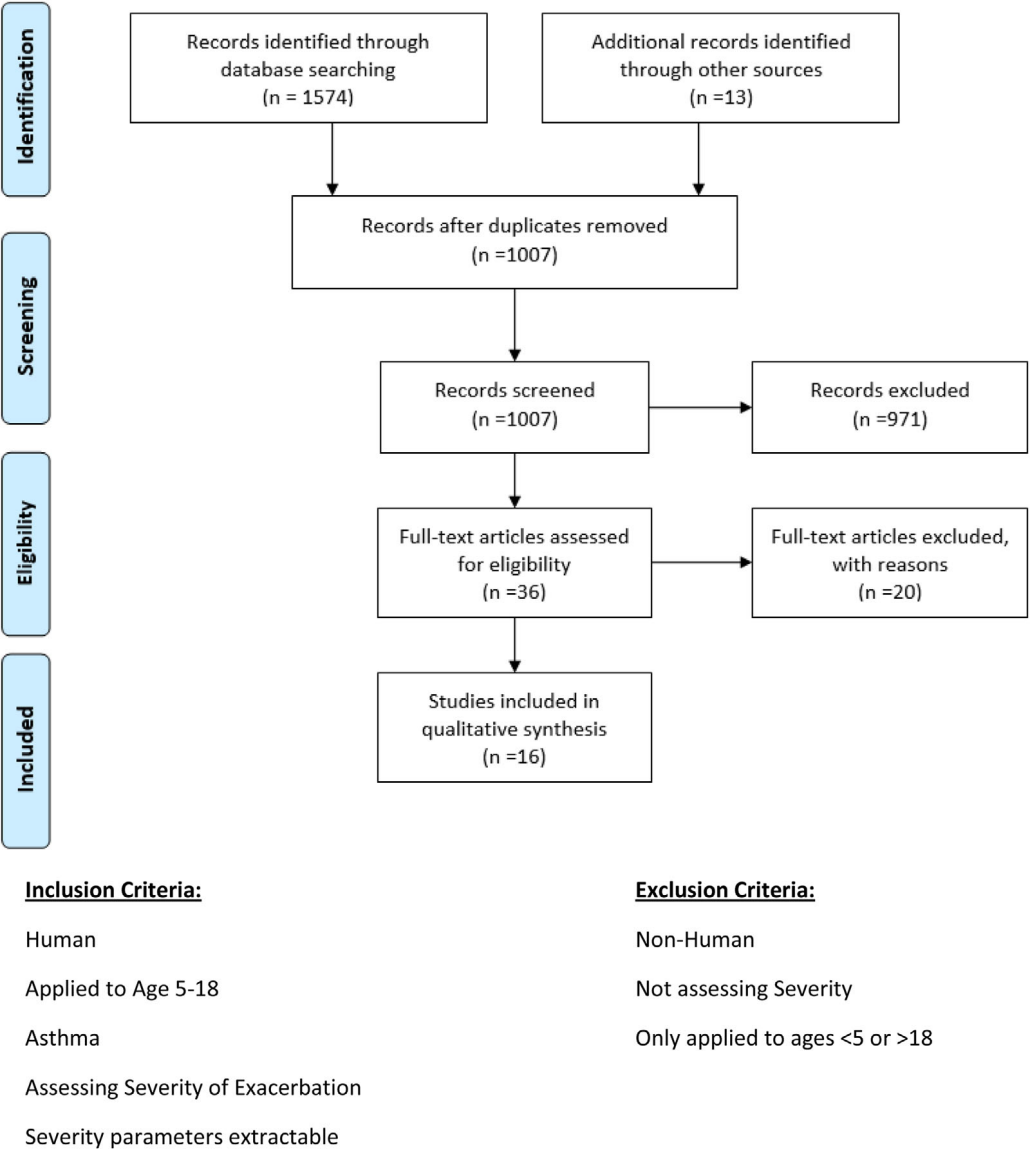
PRISMA flow diagram showing studies identified and included in the systematic review

A total of 1574 studies were identified, with an additional 13 studies obtained through additional sources. After removing duplicates, 1007 articles were screened with 36 articles undergoing full‐text screening. Of the 36 articles, 20 were excluded resulting in 16 eligible studies. Supporting Information File S4 states reasons for exclusion of full‐text articles. The search strategy is summarized in Figure 1.

Our systematic review identified 17 different asthma severity scores. Within the severity scores there were a total of 15 different parameters measured (Table 1). Severity scores assessed a median of 6 parameters (range, 4–8).

**TABLE 1:** Components included in published pediatric severity scores

Symptom/sign	Specific parameter recorded in the severity scores	MPIS^13^	Koumbourlis^14^	ASS^15^	CAS^16^	CAES2^17^	SCAS^18^	ASS adj^19^	ASS2^19^	PAS^20^	PASS^21^	AAIRS^22^	PRAM^23^	PIS^24^	RAD^25^	CS^26^	PS^27^	RA^28^
Wheeze	Inspiratory wheeze	+	+	+			+	+	+	+		+	+	+		+	+	+^a^
	Expiratory wheeze	+	+	+		+	+	+	+	+	+	+	+	+		+	+	+^a^
	Audible without stethoscope			+		+		+	+			+	+	+				
Dyspnea				+		+			+						+		+	
Rate of breathing	Respiratory rate/tachypnea	+			+		+			+	+			+	+	+	+	+
Quality of the entry of air	Aeration/air entry/breath sounds	+	+			+					+	+	+		+			+
Inhalation/exhalation length	Inhalation‐exhalation ratio/prolonged expiratory phase	+	+								+	+		+				+
Heart rate		+		+			+											
O_2_ saturation		+			+	+			+			+						
Accessory muscle use or retraction or work of breathing	General accessory muscle use/increased work of breathing	+		+		+	+	+	+		+			+				+
	Suprasternal muscle/SCM retraction		+									+	+		+		+	
	Substernal/subcostal/intercostal recession		+		+					+		+			+	+		
	Supraclavicular contractions		+		+					+						+		
	Scalene muscle retraction												+		+			
State of alertness	Cerebral function/mental status					+					+							
Total number of parameters used		8	7	5	5	5	6	5	4	7	6	7	7	6	5	6	4	6

^a^ Wheeze not specified as either inspiratory or expiratory.

The most common symptoms and/or signs measured among the severity scores were “accessory muscle use or retraction or increased work of breathing” (Table 1). Different scores required variations of this symptom to be recorded, with “general assessment of accessory muscle use or increased work of breathing” required in 53% (9/17) of the severity scores, “Intercostal/Substernal/Subcostal recession” in 35% (6/17), whereas “suprasternal/sternocleidomastoid retraction” use is recorded in 29% (5/17). Only 2 severity scores included the use of scalene muscle retraction in their assessment.

The second most commonly assessed symptom/sign used in the severity scores was wheeze, required in 88% (15/17) of severity scores. Expiratory wheeze was used in 15/17 (88%) of severity scores, 13/17 (76%) included inspiratory wheeze, 7/17 (47%) included wheeze audible without a stethoscope.

The mean number of participants per study describing each severity score was 239 (range, 30–1221). In 65% (11/17) of manuscripts, there were fewer than 100 participants.

### 3.1 PERUKI site survey

There were 59 PERUKI sites that responded to the survey from a total of 63. Responses were from consultants (53/59), nurses (3/59), and other (3/59). The responses from each center were provided by the lead clinician or a single delegate responsible for working with the PERUKI team.

From the 15 parameters in the assessment scores, the median number of these parameters collected by the PERUKI sites was 7 (range, 3–14) (Table 2). The most commonly reported signs and symptoms from PERUKI sites were O_2_ saturations (59/59), with 58/59 sites recording heart rate and respiratory rate. Supraclavicular contraction was recorded as being always captured at 1 hospital site.

**TABLE 2:** Parameters recorded at each site

Parameter recorded	Percentages of hospital sites that recorded parameter (%)	Number of centers that recorded parameter (out of 59)
0_2_ saturation	100.0	59
Heart rate	98.3	58
Respiratory rate	98.3	58
General accessory muscle use/increased work of breathing	64.4	38
Recession	55.9	33
Breath sounds	52.5	31
Tachypnea	52.5	31
Expiratory wheeze	49.2	29
Cerebral function/mental status	47.5	28
Wheeze audible without stethoscope	32.2	19
Inspiratory wheeze	25.4	15
Dyspnea	23.7	14
Substernal/subcostal/intercostal recession	18.6	11
Suprasternal muscle/SCM retraction	16.9	10
Inhalation‐exhalation ratio/prolonged expiratory phase	5.1	3
Scalene muscle retraction	3.4	2
Supraclavicular contraction	1.7	1

Using their routinely collected data, Table 3 shows which severity scores could currently be used in each center. Previously published validation for the scores are also noted in the table, based on a study that compared the validity, reliability, and use of clinical scores that assessed dyspnea.^29^ This systematic review published by Bekhof et al^29^ showed that many of the severity scores had not been sufficiently validated to be clinically significant in this cohort of children. The severity scores were rated using the following system: positive (+), intermediate (±), negative (−), unclear (?), or no information (0). The sum of the positive‐rated criteria is shown in Table 3 for the severity scores analyzed in the systematic review.

**TABLE 3:** PERUKI site and severity score with validity scores

Severity score	PERUKI sites who collect full dataset	Number of PERUKI sites compared to all PERUKI sites (%)	Average additional parameters required per site to complete severity score	Validity score^29^
ASS^15^	8	13.6	2.3	4
CAES2^17^	7	11.9	2.5	4
PIS^24^	6	10.2	3.3	5
MPIS^13^	3	5.1	3.0	4
PASS^21^	3	5.1	2.8	5
RA^28^	2	3.4	3.8	4
PS^27^	2	3.4	2.1	2
CS^26^	1	1.7	3.9	3
CAS^16^	1	1.7	2.7	5
PRAM^23^	0	0.0	4.2	5
RAD^25^	0	0.0	3.2	4

Of the 59 PERUKI sites that responded, 33.90% (20/59) stated that they used a severity scoring system in their EDs, with 11 sites saying their scoring system was based on the BTS/SIGN guidelines. Within the sites, 18 used electronic record attendances, 17 via paper, and 24 recorded asthma attendances with a combination of both paper and electronic. It is unclear if the sites have asthma assessments templates or required fields in their documentation, because this was not examined.

The severity scores ASS, ASS2, and ASS‐adj all had the highest percentage of complete collection among the PERUKI sites, with 8 sites (13.6%) reporting they always collected the required parameters. Of the 17 severity scores found among the systematic review, 3 severity scores (Koumbourlis, PRAM, and RAD) had no PERUKI sites that always collected the required parameters.

Of the 17 severity scores, 11 had validity scores associated. Among the well‐validated scores like the Pulmonary Index Score (PIS), Pediatric Asthma Severity Score (PASS), Childhood Asthma Score (CAS), and the Pediatric Respiratory Assessment Measure (PRAM), only 6/59 (10.2%), 3/59 (5.1%), 1/59 (1.7%), and 0 (0%) of units always collected the data required to calculate them.^29^ There were 6 severity scores for which we could not identify any previous published validation; these have been excluded from the analysis. Of the severity scores with a validity score, the ASS score had the highest percentage of complete collection among PERUKI sites. Both the PRAM and RAD severity score had the lowest number of PERUKI sites collecting the full dataset; however, these both scored high for validity score.

**FIGURE 2:**
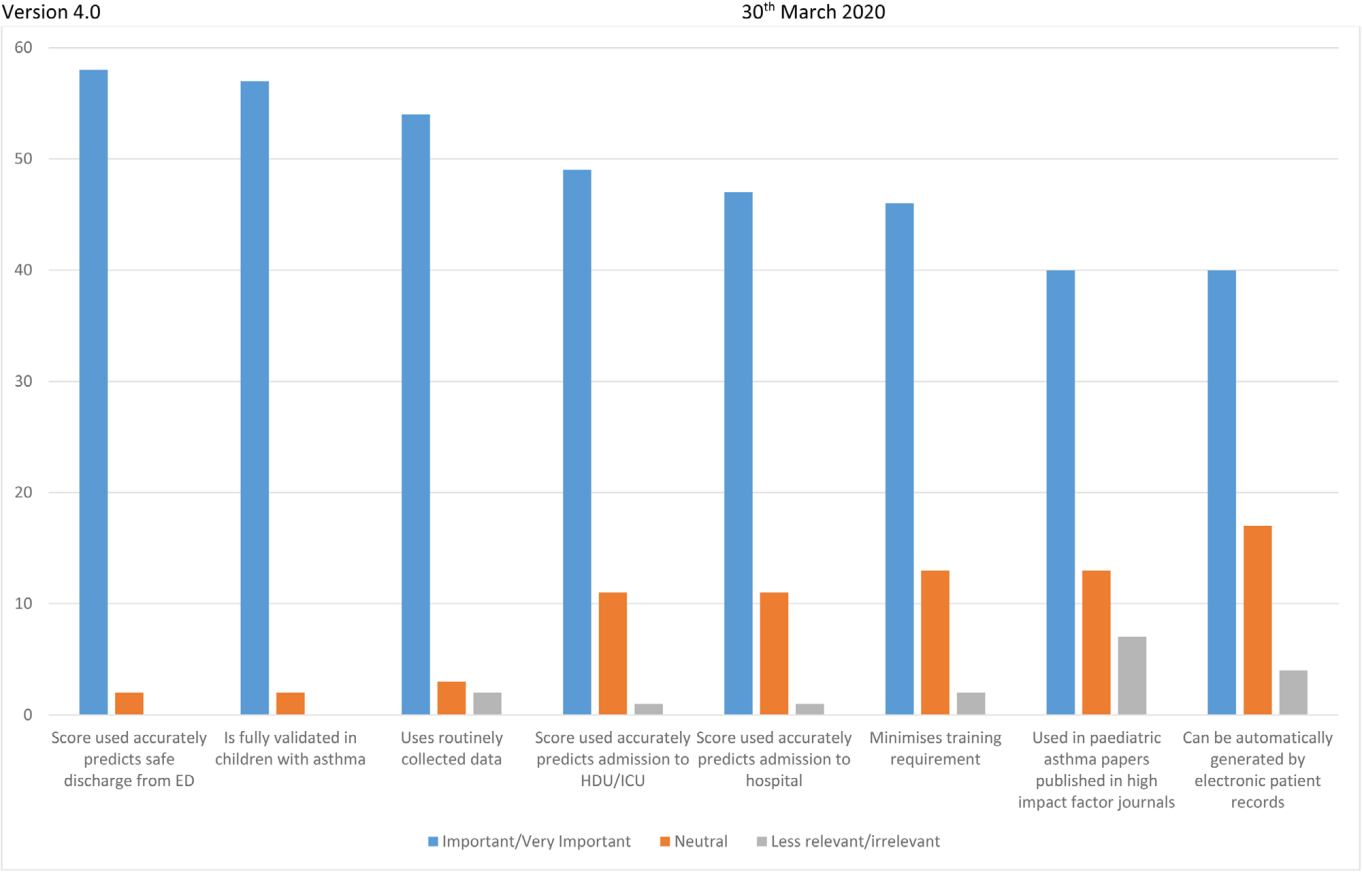
Features of a pediatric asthma severity score prioritized by PERUKI sites

The survey also asked PERUKI centers about which factors they would view as most important or useful in a PASS (see Supporting Information for full survey). These data are shown in Figure 2. The most prioritized features were “Score used accurately predicts safe discharge from ED” (58/59) and “is fully validated in children” (57/59). The features that were judged least useful by PERUKI centers were “used in pediatric asthma papers published in high impact factor journals” (40/59) and “Can be automatically generated by electronic patient records” (40/59), although all options were still prioritized by more than two out of three units (Figure 2).

## 4 LIMITATIONS

The use of a single responder in the survey could lead to responder bias, because the environment that the responder worked in, their knowledge of interpreting the parameters in children, and how they interpreted the questions being asked in the survey is unclear. In some instances a responder may have answered the question from their perception of what is captured or from first‐hand knowledge of clinical practice; unfortunately, this was not examined in the survey.

## 5 DISCUSSION

Accurate assessment of the severity of an asthma exacerbation is important to aid clinical decision‐making, audit, and research. This systematic review highlights the considerable range of published pediatric asthma severity scores that exist and how they all use combinations of a common set of 15 parameters to generate their scores.

The optimal severity score to use when children and young people attend with an acute exacerbation of asthma will depend on several factors. Key areas that need to be considered include the use of the score (safety of discharge, risk of PICU admission, something else), accuracy, whether or not it is validated in children, and whether or not clinical teams need additional training or resources to capture the required parameters.

These questions require additional research to answer, and to date, there has been little comparative research between the various pediatric asthma severity scores. To facilitate this research, there will need to be improvements in routine data collection. The majority of the parameters required for existing asthma severity scores are already included in the routine clinical assessment of a child with an asthma attack. The variability is therefore likely to be in the recording these data. Our survey shows that, currently, even if units wished to use these scores, they would not be able to derive them.

Electronic patient records may offer a way to improve this, because there are options to have mandatory fields, to ensure the recording of data, thereby generating large datasets where multiple severity scores can be calculated and compared. Implementing patient pathways for children's asthma supported by electronic patient records may be beneficial, as it has helped deliver improvements in outcomes for other conditions.^30^

For the vast majority of the PERUKI respondents who represent the pediatric ED teams seeing children with acute asthma, it was a priority that any severity score be validated in children. Validity in the pediatric population has been considered previously but only included a sub‐set of the severity scores identified in this work.^29^

Training was identified as another important issue. Scalene muscle retraction was very poorly recorded in the survey, but is a requirement in 2 of the severity scores.^23^, ^25^ This parameter is dependent on palpation, and to improve the use of this, there would be a considerable investment in training required.

Although most of the children and young people who attend EDs with acute asthma fully recover, around 20 children a year in the United Kingdom die from acute asthma attacks.^31^ There is a pressing need to understand how to identify and optimally manage this population. Improved data collection would only represent one aspect of this, but if it were achieved, then the delivery of acute asthma research would be simplified and research beyond the ED (eg, into the striking regional variation in asthma attendances) would be facilitated.

## 6 CONCLUSION

Standardized PASS are infrequently used in the United Kingdom and Ireland, although individual parameters are often collected by EDs. Improved routine data collection focusing on the key parameters common to multiple scores could improve this, facilitating research and audit of pediatric acute asthma.

## COLLABORATORS

The following people acted as PERUKI site lead investigators and were responsible for coordinating local processes and submission of data: Matt Rotheram (Alder Hey NHS Foundation Trust); Caroline Ponmani (Barking Havering and Redbridge University Trust); Stuart Hartshorn (Birmingham Children's Hospital); Mark D. Lyttle (Bristol Royal Hospital for Children); Adrian Boyle (Cambridge University Hospitals Foundation Trust); Carolyn Hore (Chelsea and Westminster NHS Foundation Trust); Roisin McNamara (Children's Health Ireland at Temple Street); Turlough Bolger (Children's Health Ireland at Tallaght University Hospital); Michael Barrett (Children's Health Ireland at Crumlin); Rory O'Brien (Cork University Hospital); Chris Vorwerk (Queen Alexandra Hospital, Portsmouth); Adama Kargbo (Royal Free Hospital, London); Godfrey Nyamugunduru (County Durham and Darlington NHS Foundation Trust); Sylvester Gomes (Evelina London Children's Hospital); Elizabeth Herrieven (Hull University teaching Hospitals NHS Trust); David Hartin (Ipswich Hospital, East Suffolk and North Essex NHS Foundation Trust); Arshid Murad (James Cook University Hospital, Middlesbrough); Chris Bird (John Radcliffe Hospital, Oxford University Hospitals NHS Foundation Trust); Fleur Cantle (Kings College Hospital, London); Kirsty Challen (Lancashire Teaching Hospitals NHS Trust); Alice Downes (Leeds General Infirmary); Damian Roland (Leicester Royal Infirmary); Adebayo Da'Costa (Medway NHS Foundation Trust); Nicola Keane (Medway NHS Foundation Trust); Clare Dieppe (Morriston Hospital, Swansea Bay University Health Board); Esther Wilson (Musgrove Park Hospital, Taunton); Mark Anderson (Newcastle upon Tyne Hospitals NHS Foundation Trust); Dermot Dalton (North Devon District Hospital); Dianne Cook (North Manchester Care Organisation); Charlotte Clements (North Middlesex University Hospital NHS Trust); John Gibbs (Nottingham University Hospitals NHS Trust); Sharon Hall (Queen Elizabeth Hospital Woolwich); Gareth Patton (Royal Aberdeen Children's Hospital); Emily Walton (Royal Alexandra Children's Hospital, Brighton); Julie‐Ann Maney (Royal Belfast Hospital for Sick Children); Manish Thakker (Royal Berkshire NHS Foundation Trust); Andy Appelboam (Royal Devon and Exeter Hospital NHS Foundation Trust); Steven Foster (Royal Hospital for Children, Glasgow); Jen Browning (Royal Hospital for Sick Children, Edinburgh); Katherine Potier (Royal Manchester Children's Hospital); Elizabeth Gilby (Royal United Hospital, Bath); Seb Gray (Salisbury NHS Foundation Trust); Shammi Ramlakhan (Sheffield Children's Hospital NHS Foundation Trust); Niall Mullen (South Tyneside and Sunderland NHS Foundation Trust); Sharryn Gardner (Southport and Ormskirk NHS Trust); Heather Jarman (St George's University Hospitals NHS Foundation Trust); Neil Thompson (St Mary's Hospital, Imperial College Healthcare NHS Trust); Ami Parikh (The Royal London Hospital); Lisa Kehler (The Royal Wolverhampton NHS Trust); Penny Salt (University College London Hospital); Joanne mulligan (University Hospital Crosshouse); Sophie Keers (University Hospital Lewisham); Jeff Morgan (University Hospital of Wales); David James (University Hospital Southampton); Gisela Robinson (University Hospitals of Derby and Burton NHS Foundation Trust); Andy Robinson (University Hospitals Plymouth NHS Trust); Lynn Sinitsky (Watford General Hospital, West Herts NHS Trust); Mike Linney (Western Sussex NHS Foundation Trust).

## CONFLICT OF INTEREST

The authors have no conflict of interest to disclose.

## Supporting information

Supporting information
