# Supplementary material for: Pediatric acute asthma scoring systems: a systematic review and survey of UK practice
Source: J Am Coll Emerg Physicians Open. 2020 Jun 2;1(5):1000–8. doi: 10.1002/emp2.12083 (PMC7593416; doi:10.1002/emp2.12083)
Supplement: Supplementary file 1 — Supporting information [file EMP2-1-1000-s001.docx]

Paediatric Acute Asthma Scoring Systems: A Systematic Review and Survey of UK Practice (Supplementary data section

Jerry Chacko^1,2^, Charlotte King^3^, David Harkness^4^, Shrouk Messahel^5^, Julie Grice^5^, John Roe^6^, Ian P. Sinha^7^, Daniel B Hawcutt^2^ on behalf of PERUKI

**1:** School of Medicine, University of Liverpool, Liverpool

**2:** Department of Women's and Children's Health, Institute of Translational Medicine, University of Liverpool, Liverpool

**3:** Royal Liverpool and Broadgreen University Hospital Trust, Liverpool

**4:** National Institute for Health Research Alder Hey Clinical Research Facility, Alder Hey Children’s Hospital, Liverpool

**5:** Emergency Department, Alder Hey Children’s Hospital, Liverpool

**6:** Darwin Emergency Department, Australia

**7:** Department of Respiratory Medicine, Alder Hey Children’s Hospital, Liverpool

# Methods

## Systematic Review Search Terms

(Paediatric:)

“paediatric*” OR “pediatric*” OR “peadiatric*” OR “infant*” OR “infanc*” OR “adolecen*” OR “juvenile*” OR “child*” Or “children*” OR “kid*” OR “teen*” OR “boy*” OR “girl*” OR “young” OR “school” OR “schoolchild*” OR “school age*” OR “preschool*” OR “pre-school*” OR “toddler*” OR “primary school*” OR “elementary school*” OR “secondary school*” OR “high school*”

AND

(Asthma:)

“asthma*” OR “wheez*” OR “bronchial hyperresponsiveness*” OR “bronchial hypersensitive*” OR “bronchial hyperresons*” OR “difficulty breathing*” OR “shortness of breath” OR “airway difficulty*” OR “airway obstruct*” OR “dyspe*” OR “chest tight*” OR “airway inflammation*” OR “respiratory obstruct*” OR “respiratory hypersensit*”

AND

(Severity Score:)

"severity score" OR "scoring system" OR "symptom score" OR "illness score" OR “pulmonary index” OR “pulmonary score”

## Systematic Review Inclusion and Exclusion Criteria

**Inclusion Criteria:**

Human

Applied to Age 5-18

Asthma

Assessing Severity of Exacerbation

Severity parameters extractable

**Exclusion Criteria:**

Non-Human

Not assessing Severity

Only applied to ages <5 or >18

### Survey sent to PERUKI sites

1. Which Emergency Department do you work in?
2. Role in the Emergency Department?
3. Which of the following data are ALWAYS captured in the ED for children aged 5-18 who present with exacerbation of asthma? Please include all triage, nursing and medical assessments that are mandated on electronic systems, pathways, and/or clinical documentation. Select ALL that apply
   1. Inspiratory Wheeze
   2. Expiratory Wheeze
   3. Wheeze audible without stethoscope
   4. Dyspnoea
   5. Heart Rate
   6. O2 saturations
   7. Respiratory Rate/Tachypnoea
   8. Aeration/Air entry/Breath sounds
   9. Inhalation-exhalation ratio/Prolonged expiratory phase
   10. General accessory muscle use/Increased work of breathing
   11. Suprasternal muscle/SCM retraction
   12. Substernal/subcostal recession/Intercostal recession
   13. Scalene Muscle Retraction
   14. Supraclavicular Contraction
   15. Cerebral function/mental status
4. Does your ED currently use a severity score for children with asthma?
   1. YES/NO
   2. Please specify if one used
5. Does your ED record asthma attendance on paper or electronically?
   1. Electronic
   2. Paper
   3. Combination of paper and electronic
6. Which oral steroid does your department use for patients with acute exacerbation of asthma?
   1. Prednisolone
   2. Dexamethasone
   3. Other
7. What would you consider most important/ useful when selecting an asthma severity scoring system in your ED?
   1. Uses routinely collected data
   2. Used in paediatric asthma papers published in high impact factor journals
   3. Minimises training requirement
   4. Can be automatically generated by electronic patient records
   5. Is fully validated in children with asthma
   6. Score used accurately predicts admission to hospital
   7. Score used accurately predicts safe discharge from ED
   8. Score used accurately predicts admission to HDU/ICU
8. Any other comments about asthma severity in children?

| Title | Author and Year | Reason for Exclusion |
| --- | --- | --- |
| An English and Spanish pediatric asthma symptom scale | Lara 2000 | Does not assess severity of exacerbation |
| Correlation of Pediatric Asthma Severity Score and End Tidal CO2 Values With Asthma Severity in the Pediatric Population | Dhillon 2009 | Does not contain new severity score |
| Evaluation of an asthma severity score | Yung 1996 | Does not contain new severity score |
| Is pulmonary index score suitable for the evaluation of paediatric acute asthma? | Saretta 2011 | Does not contain new severity score |
| Modified Pulmonary Index Score Was Sufficiently Reliable to Assess the Severity of Acute Asthma Exacerbations in Children | Maekawa 2014 | Does not contain new severity score |
| Predicting Hospitalization In Children With Acute Asthma | Buyuktiryaki 2013 | Does not contain new severity score |
| Prospective Evaluation of Two Clinical Scores for Acute Asthma in Children 18 Months to 7 Years of Age | Gouin 2010 | Does not contain new severity score |
| Systematic review: Insufficient validation of clinical scores for the assessment of acute dyspnoea in wheezing children | Bekhof 2014 | Does not contain new severity score |
| The pulmonary index score as a clinical assessment tool for acute childhood asthma | Hsu 2010 | Does not contain new severity score |
| Usefulness of modified Pulmonary Index Score (mPIS) as a quantitative tool for the evaluation of severe acute exacerbation in asthmatic children | Koga 2015 | Does not contain new severity score |
| A simplified severity score for acute asthma exacerbation | Dankner 2013 | Does not meet age criteria |
| Clinical Scores for Dyspnoea Severity in Children: A Prospective Validation Study | Eggink 2016 | Does not meet age criteria |
| Development of a clinical asthma score for use in hospitalized children between 1 and 5 years of age | Parkin 1996 | Does not meet age criteria |
| Transcutaneous Oxygen and Carbon Dioxide Levels and a Clinical Symptom Scale for Monitoring the Acute Asthmatic State in Infants and Young Children | Wennergren 1986 | Does not meet age criteria |
| Relationship between arterial blood gas tensions and a clinical scoring system in asthmatic patients Japanese | Obata 1992 | Language not available- Japanese |
| Relationship Between Arterial Blood-Gas Tensions And A Clinical Score In Asthmatic-Children | Obata 1992 | Language not available- Japanese |
| A clinical scoring system for the diagnosis of respiratory failure Preliminary report on childhood status asthmaticus | Wood 1972 | No Full Paper Available |
| The asthma clinical score and oxygen saturation | Dawson 1991 | Does not contain new severity score |
| The usefulness of a clinical scoring system and pulse oximetry (SaO2) in assessing the severity of asthmatic crises Spanish | Luaces 1996 | No Full Paper Available |
| AAIRS Score Overview: The Acute Asthma Intensity Research Score | Arnold 2015 | Severity not assessed. Description of severity score only. |
